# A Meta-Analysis of *Wolbachia* Transcriptomics Reveals a Stage-Specific *Wolbachia* Transcriptional Response Shared Across Different Hosts

**DOI:** 10.1534/g3.120.401534

**Published:** 2020-07-24

**Authors:** Matthew Chung, Preston J. Basting, Rayanna S. Patkus, Alexandra Grote, Ashley N. Luck, Elodie Ghedin, Barton E. Slatko, Michelle Michalski, Jeremy M. Foster, Casey M. Bergman, Julie C. Dunning Hotopp

**Affiliations:** * Institute for Genome Sciences, University of Maryland School of Medicine, Baltimore, MD 21201; † Department of Microbiology and Immunology, University of Maryland School of Medicine, Baltimore, MD 21201; ‡ Institute of Bioinformatics, University of Georgia, Athens, GA 30602; § Department of Biology, Center for Genomics and Systems Biology, New York University, NY 10003; ** New England Biolabs, Ipswich, MA 01938; †† Department of Biology, University of Wisconsin Oshkosh, WI 54901; ‡‡ Department of Genetics, University of Georgia, Athens, GA 30602; §§ Greenebaum Cancer Center, University of Maryland, Baltimore, MD 21201

**Keywords:** *Wolbachia*, transcriptomics, RNA-Seq, meta-analysis, intracellular, bacteria

## Abstract

*Wolbachia* is a genus containing obligate, intracellular endosymbionts with arthropod and nematode hosts. Numerous studies have identified differentially expressed transcripts in *Wolbachia* endosymbionts that potentially inform the biological interplay between these endosymbionts and their hosts, albeit with discordant results. Here, we re-analyze previously published *Wolbachia* RNA-Seq transcriptomics data sets using a single workflow consisting of the most up-to-date algorithms and techniques, with the aim of identifying trends or patterns in the pan-*Wolbachia* transcriptional response. We find that data from one of the early studies in filarial nematodes did not allow for robust conclusions about *Wolbachia* differential expression with these methods, suggesting the original interpretations should be reconsidered. Across datasets analyzed with this unified workflow, there is a general lack of global gene regulation with the exception of a weak transcriptional response resulting in the upregulation of ribosomal proteins in early larval stages. This weak response is observed across diverse *Wolbachia* strains from both nematode and insect hosts suggesting a potential pan-*Wolbachia* transcriptional response during host development that diverged more than 700 million years ago.

*Wolbachia* endosymbionts are obligate intracellular bacteria found in many different arthropods and nematodes (Werren *et al.* 2008; Landmann 2019; Slatko *et al.* 2014). Arthropod *Wolbachia* are not typically required for their host’s survival and frequently exert a form of reproductive parasitism on their host (Engelstadter and Telschow 2009; Cordaux *et al.* 2011). In contrast, *Wolbachia*-infected filarial nematodes require their *Wolbachia* endosymbionts for survival, with *Wolbachia* depletions causing defects in proper nematode development and reproduction, eventually leading to host death (Slatko *et al.* 2010; Landmann *et al.* 2011; Taylor *et al.* 2010). As such, antibiotics can be used to treat nematode diseases such as lymphatic filariasis and onchocerciasis (Taylor *et al.* 2014; Taylor *et al.* 2005; Clare *et al.* 2019; Hong *et al.* 2019; von Geldern *et al.* 2019; Jacobs *et al.* 2019; Taylor *et al.* 2019).

Because of the considerable impact of *Wolbachia* on their hosts’ evolution, physiology, and development, combined with their intractability to many experimental approaches as obligate intracellular bacteria, ’omics-based studies have been conducted to interrogate the symbiotic relationships between *Wolbachia* and their hosts (Landmann 2019; Darby *et al.* 2012; Luck *et al.* 2014; Grote *et al.* 2017; Chung *et al.* 2019; Luck *et al.* 2015; Gutzwiller *et al.* 2015; Darby *et al.* 2014). In 2012, the first *Wolbachia* transcriptomics study assessed gene expression in the *Wolbachia* endosymbiont of the filarial nematode *Onchocerca ochengi*, *w*Oo, in adult male and female worms (Darby *et al.* 2012). Similar studies have focused on other filarial nematodes, with the transcriptomes of the *Wolbachia* endosymbionts of both *Dirofilaria immitis* (Luck *et al.* 2014) and *Brugia malayi* (Grote *et al.* 2017; Chung *et al.* 2019) being interrogated at various points of the nematode life cycle. The *w*Di transcriptome has also been assessed in different body tissues of *D. immitis* (Luck *et al.* 2015). For arthropod *Wolbachia*, transcriptomics approaches have been used to interrogate the *Wolbachia* endosymbiont of *Drosophila melanogaster*. One study assessed the transcriptome of *w*Mel in the embryo, larvae, pupae, adult male, and adult female life stages of *D. melanogaster* (Gutzwiller *et al.* 2015) while another study assessed the transcriptome of *w*MelPop-CLA cultured in a mosquito cell line in response to doxycycline treatment (Darby *et al.* 2014).

However, there is unexpected discordance among *Wolbachia* transcriptomics studies, as the different studies identify varying numbers of differentially expressed genes, despite having similar samples being assessed. As the first *Wolbachia* transcriptomics study was published in 2012 (Darby *et al.* 2012), this discordance could be attributed to a difference in sequencing technologies and analysis tools over the past 8 years. Prior to the development of enrichment techniques, such as targeted RNA-Seq captures (Martin *et al.* 2016; Chung *et al.* 2018b), older *Wolbachia* transcriptomics studies may not have been able to sequence *Wolbachia* to a sufficient depth for differential expression analyses. Additionally, the more recent application of saturation curves to RNA-Seq analyses has proven to be a robust method to assess whether samples have been sequenced to a sufficient depth (Sims *et al.* 2014) and dual-species transcriptomics studies benefit from the implementation of a rigorous minimum expression filter. Older differential expression tools also had to rely on multiple pairwise comparisons to identify differentially genes while the current iteration of differential expression tools, such as DESeq2 (Love *et al.* 2014) and edgeR (Robinson *et al.* 2010), are able to test 3 or more conditions simultaneously. Here, we used all available *Wolbachia* transcriptomics data sets to perform a large-scale transcriptomics meta-analysis with a unified pipeline with the best practices of current transcriptomics analysis methods, with the goal of identifying conserved patterns of *Wolbachia* differential expression across diverse taxa.

## Methods

### Alignments and quantification

SRA data were downloaded from each of the seven studies using SRA-Toolkit v2.9 (Leinonen *et al.* 2011) with the SRP IDs as shown in (Table S1). For each study, HISAT2 v2.1.0 (Kim *et al.* 2015) was first used to map reads to the genomes of the *Wolbachia* endosymbiont and their host simultaneously (Table 2) to account for lateral gene transfer reads in the sequencing data set. Using Seqtk v1.2 (https://github.com/lh3/seqtk), reads that mapped to the genome of the subset *Wolbachia* endosymbiont were extracted from the initial FASTQ file and mapped against both genomes again using HISAT2 v 2.1.0 (Kim *et al.* 2019) with no spliced mappings allowed and a maximum fragment size of 1 kbp. The resulting alignment files were quantified using FADU v1.4 (Chung *et al.* 2018a) with study-specific stranded options, CDS as the feature type, ID as the attribute ID, and all other settings left as default.

**Table 1:** Summary of re-analysis results across prior *Wolbachia* transcriptomics studies

Study	PMID	*Wolbachia strain*	Host Species	Wolbachia Reads Mapped	Wolbachia Reads Mapped to Coding Sequences	Samples Excluded from Saturation Analysis	DE Genes in Original Study	DE Genes in Reanalysis	Core DE Genes in Reanalysis
Darby *et al.* 2012	22919073	*wOo*	*Onchocerca ochengi*	71,457-416,870	26,067-146,642	0/4	26	0	0
Luck *et al.* 2014	25433394	*wDi*	*Dirofilaria immitis*	16,372-328,230	495-27,118	3/5	653	NA	NA
Darby *et al.* 2014	24152719	*wMelPop-CLA*	*Aedes albopictus* cell line RML-12	3,267,276-13,265,718	42,960-120,344	0/6	78	120	72
Luck *et al.* 2015	26559510	*wDi*	*Dirofilaria immitis*	190-437,866	0-348	6/12	33	36	35
Gutzwiller *et al.* 2015	26497146	*wMel*	*Drosophila melanogaster*	138,932-10,914,210	18,862-417,013	0/55	80	473	325
Grote *et al.* 2017	28358880	*wBm*	*Brugia malayi*	572,700-2,746,978	6.178-31,289	0/14	62	94	83
Chung *et al.* 2019	31796568	*wBm*	*Brugia malayi*	117,194-82,996,546	8,525-35,276,890	0/32	318	373	338

**Table 2:** Reference genomes used for meta-analysis

Organism	Reference Version
*Brugia malayi*	WormBase: WS270
*Dirofilaria immitis*	nematodes.org: nDi 2.2
*Drosophila melanogaster*	FlyBase: r6.27
*Onchocerca ochengi*	nematodes.org: nOo 2.0
Wolbachia endosymbiont of *Brugia malayi*, *w*Bm	RefSeq: NC_006833.1
Wolbachia endosymbiont of *Dirofilaria immitis*, wDi	nematodes.org: *w*Di 2.2
Wolbachia endosymbiont of *Drosophila melanogaster*, *w*Mel	RefSeq: NC_002978.6
Wolbachia endosymbiont of *Onchocerca ochengi*, *w*Oo	RefSeq: NC_018267.1

### Transcriptomics analyses

Transcriptomic analyses were done using R v3.5.0. Rarefaction plots were generated using the R package vegan v2.5-4 (Dixon 2003) using the CDS counts derived from FADU as an input. Principal component analyses were conducted using the R package FactoMineR v 1.41 (Lê *et al.* 2008) with log_2_ TPM values. Dendrograms were constructed using the R packages dendextend v1.9 (Galili 2015), ggdendro v0.1-20 (de Vries and Ripley 2016), and pvclust v2.0-0 (Suzuki and Shimodaira 2006) using the z-score of log_2_ TPM. The R packages cowplot v0.9.4 (Wilke 2019), gplots v3.0.1.1 (Warnes *et al.* 2019) and ggplot2 v3.1.1 (Wickham 2016) were used to create all other figures. Functional terms describing the functional characteristics of each gene such as gene ontology (GO) terms and InterPro descriptions were obtained using InterProScan v5.34-73 (Jones *et al.* 2014) and the nucleotide coding sequences for each gene.

Differential expression analyses were conducted using edgeR v3.24.0 (Robinson *et al.* 2010) using a quasi-likelihood fit test (Lund *et al.* 2012; Chen *et al.* 2016), with significantly differentially expressed genes being defined as having a FDR < 0.05. Prior to differential expression analysis, genes were filtered based on the edgeR recommended minimum expression value criteria such that a gene has to have a CPM value ≥5 reads per gene in the smallest library size sample in a number of samples greater than or equal to the smallest replicate size (Robinson *et al.* 2010). WGCNA v 1.6.6 (Langfelder and Horvath 2008) was used for clustering differentially expressed genes based on expression patterns. Expression modules were derived from each dataset by hierarchically clustering genes based on dissimilarity in a topological overlap matrix with a dynamic tree cut at a height that encompasses 99% of the truncated height range in the observed dendrogram (minimum cluster size: 1). Closely related modules were merged using a merge eigengene dissimilarity threshold of 0.25. Each individual expression module was further divided into two clusters depending on whether the expression pattern of a gene has a higher Pearson correlation to the eigengene or the inverse eigengene. Fisher’s exact test was used to identify over-represented functional terms in subsets of genes from WGCNA modules and differential expression analyses (FDR < 0.05).

### Core gene analyses

Core *Wolbachia* genes between the *w*Bm, *w*Di, *w*Mel, and *w*Oo genomes were identified using PanOCT v3.23 with *-S N-M Y -H Y -F 1.33 -c 0,25,50,75,100 -T* and all other settings set to default. UpSet analyses using the core differentially expressed genes from studies in this analysis were generated using the R package UpSetR v1.4.0 (Conway *et al.* 2017). Fisher’s exact test was used to identify over-presented functional terms in each UpSet category (FDR < 0.05).

### Data availability

Figure S1 illustrates the TPM values for Luck *et al.*, 2015, for different gene biotypes. For each sample in the Luck *et al.*, 2015 study, a stacked bar was generated to display the TPM values assigned to coding sequences (red), rRNAs (green), and the 6S RNA (blue). Figure S2 illustrates the difference in the log_2_-fold change values of differentially expressed genes identified in the original and re-analysis of the Gutzwiller *et al.*, 2015 data set. Table S1 contains meta-data on each of the *Wolbachia* studies in the re-analysis, Table S2 contains the *Wolbachia* read counts per gene from each study. Table S3 contains the *Wolbachia* TPM values from each study. Table S4 contains the *Wolbachia* TPM values of differentially expressed genes and WGCNA gene assignments from each study. Table S5 contains the functional term enrichment analyses for each WGCNA expression module. Table S6 contains a list of orthologs identified between the *w*Bm, *w*Di, *w*Mel, and *w*Oo genomes. Table S7 contains the functional term enrichment analyses for each UpSet category analyzed in Figure 9. Supplementary data files and source code for reproducing the transcriptomics analyses and figures can be downloaded from Github at https://github.com/Dunning-Hotopp-Lab/A-meta-analysis-of-Wolbachia-transcriptomics-reveals-a-stage-specific-Wolbachia-transcriptional-resp (https://doi.org/10.5281/zenodo.3726272). All code is made available under the MIT License. Supplemental material available at figshare: https://doi.org/10.25387/g3.12605408.

## Results

### Overview of data sets and methods

**Figure 1:**
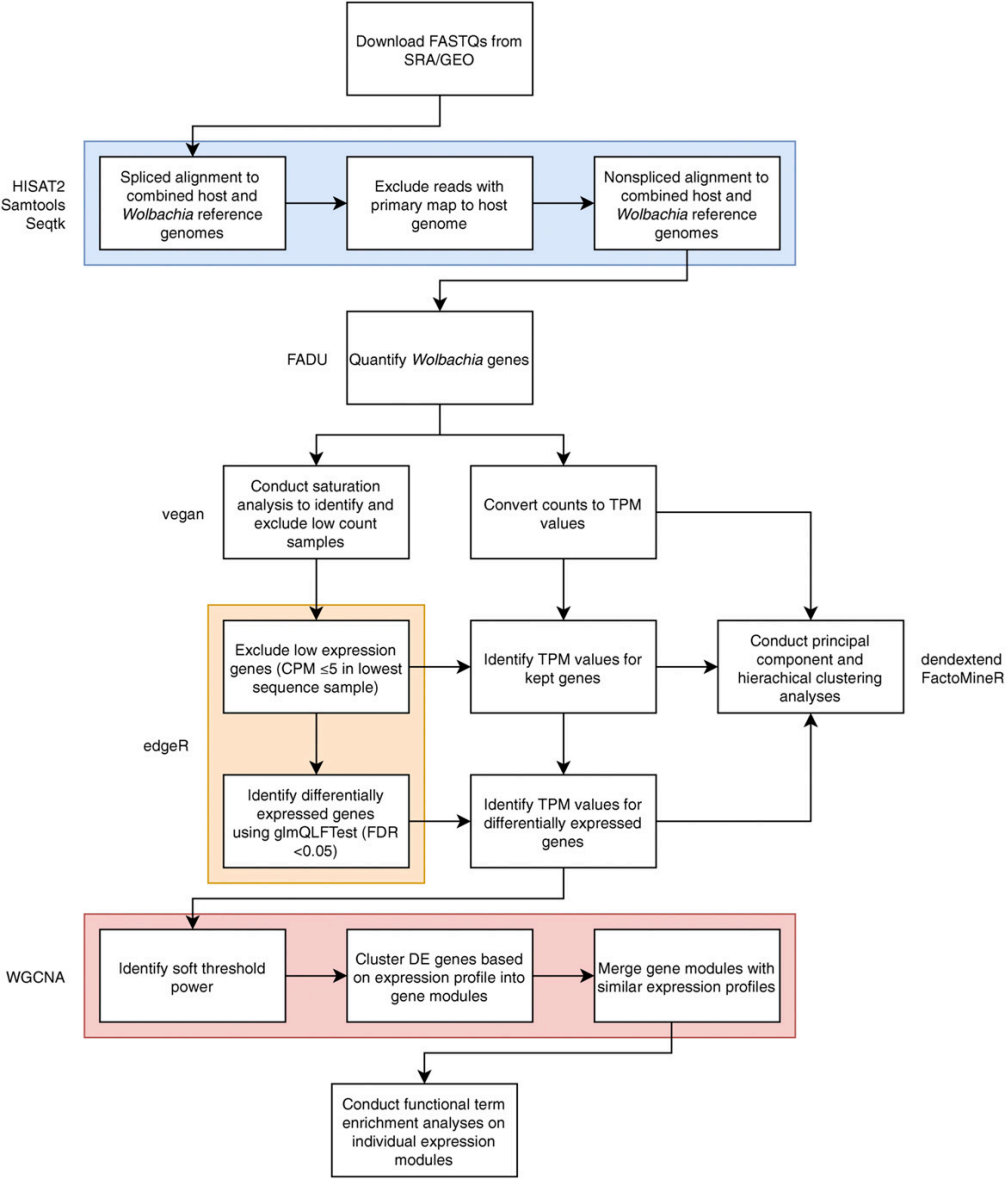
Unified workflow used for transcriptomics re-analysis For each study, reads were downloaded from their respective SRA or GEO databases and re-analyzed using the shown unified workflow including the alignment of reads to the combined host and *Wolbachia* references (blue), differential expression analysis (yellow), and the downstream clustering of differentially expressed genes based on expression profile (red). Tools used for each of the different steps are noted next to their respective boxes.

There are currently seven major published analyses of the *Wolbachia* transcriptome, with five of the studies focusing on filarial nematode *Wolbachia* and the other two focusing on *Wolbachia* strains from *D. melanogaster* (Darby *et al.* 2012; Darby *et al.* 2014; Luck *et al.* 2014; Luck *et al.* 2015; Gutzwiller *et al.* 2015; Grote *et al.* 2017; Chung *et al.* 2019). Within these seven studies, five analyze the transcriptome at different points in their host’s life cycle, one analyzes the *Wolbachia* transcriptome in different tissues of their host, and one analyzes the *Wolbachia* response to the drug doxycycline (Table 1, Table S1). To identify potential *Wolbachia* transcriptional patterns, we systematically re-analyzed all seven of these *Wolbachia* data sets using a unified RNA-Seq analysis pipeline (Figure 1).

Unlike most other bacteria, including obligate intracellular endosymbionts, *Wolbachia* are known to transfer large amounts of their DNA to the genome of their hosts (Dunning Hotopp *et al.* 2007; Dunning Hotopp 2011) with the potential for transcription in the eukaryotic host that includes splicing. If reads were only mapped in a non-splice-aware manner, host lateral gene transfer reads that would better map to the host genome in a splice-aware manner could be erroneously attributed to the *Wolbachia*, inflating the count of *Wolbachia* genes containing lateral gene transfer events (LGTs). Similarly, if only splice-aware mapping was used, erroneous spliced mappings could occur in the *Wolbachia* genome. While rarely an issue when both reference genomes are high quality, many of the nematode reference genomes are draft genomes and as such, there are frequently genome assembly issues involving LGTs. To alleviate these issues, we used a two-step mapping approach for this meta-analysis. The first mapping step maps all reads in a splice-aware manner to a combined reference containing both the *Wolbachia* and its host’s genome (Table 2). By doing this, reads originating from the eukaryotic host can be properly mapped to their genome. Next, to exclude all lateral gene transfer reads and ensure that all reads mapping to the *Wolbachia* genome are properly mapped in a non-spliced manner, we extracted all reads with a primary match to the *Wolbachia* genome and re-mapped them against the combined reference without allowing for spliced mapping, such that *Wolbachia* reads can be properly mapped to their genome. While there are potential issues of losing signal with this analysis, any over-exclusion of data will occur equally across all samples for similar reads. We also predict that this is the most conservative analysis with respect to the sequences that are shared between the endosymbiont and its host. In nematodes where there has been significant sequence divergence of LGT events this mapping strategy will work better than for insect LGT events which are known to have little sequence diversity (*e.g.*, *Drosophila ananassae*). The only insect analyzed here is *Drosophila melanogaster*, which is not known to have any LGTs (Richardson *et al.* 2012).

The resulting alignments were used to quantify read counts in coding sequences with FADU (Chung *et al.* 2018a). Differentially expressed genes were identified using edgeR (Robinson *et al.* 2010) with a quasi-likelihood fit model (Lund *et al.* 2012; Chen *et al.* 2016) and a false discovery rate (FDR) < 0.05. Each set of differentially expressed genes were then clustered using WGCNA (Langfelder and Horvath 2008) and individual expression modules were examined for over-represented GO functional terms and InterPro descriptions.

### Darby *et al.*, 2012: Sex-specific host differences in the wOo transcriptome

The study sought to identify transcriptomic differences between two biological replicates of adult male *Onchocerca ochengi* worms and gonads microdissected from adult female *O. ochengi* worms (Darby *et al.* 2012). Double-stranded cDNA was made from total RNA and used for sequencing with SOLiD (version 4; Applied Biosystems) as 50 bp color-space reads (Darby *et al.* 2012). Reads were originally mapped using Bowtie (Langmead 2010) and transcripts were quantified using Cufflinks (Trapnell *et al.* 2012), with differentially expressed genes being identified using the tools bam2rpkm (http://bam2rpkm.sourceforge.net/) and edgeR (FDR < 0.05) (Robinson *et al.* 2010) with a negative binomial distribution model. Approximately 96% of *w*Oo genes had similar expression levels between the two samples, but 26 genes were significantly upregulated in the adult female gonad samples that were associated with translation, DNA replication, and membrane transport (Darby *et al.* 2012).

**Figure 2:**
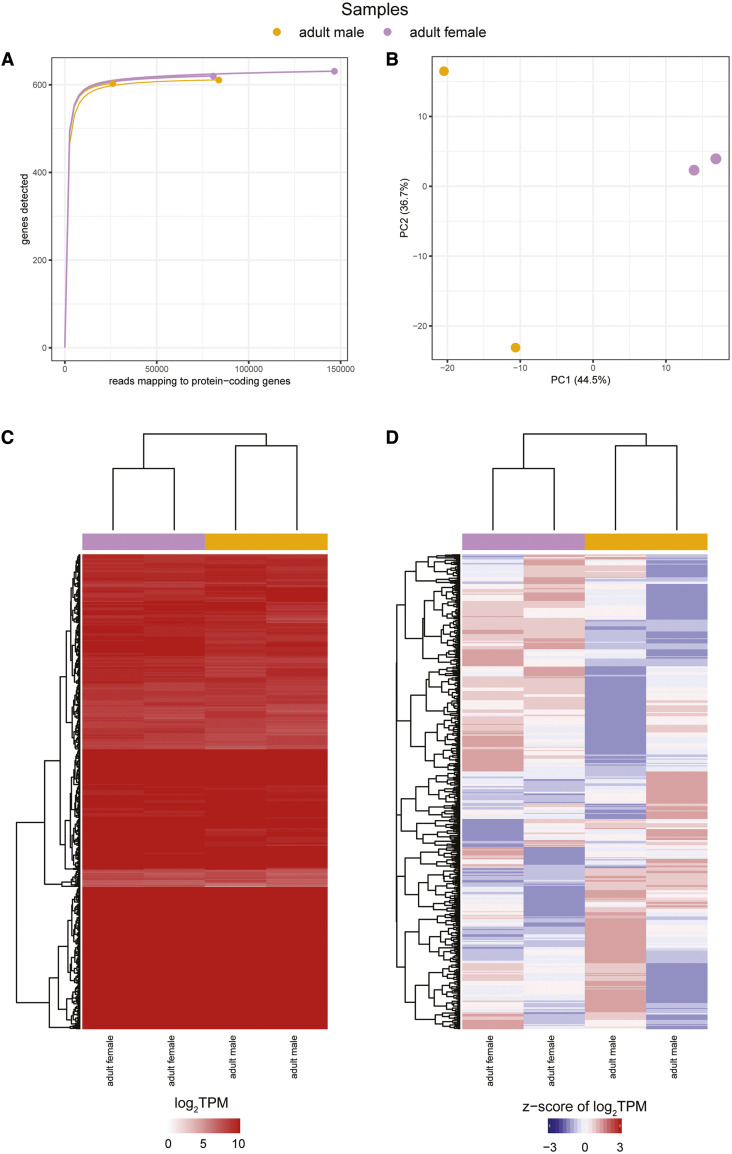
A re-analysis of the Darby *et al.*, 2012 *w*Oo transcriptome study (a) Rarefaction curves were calculated by determining the number of protein-coding genes detected from a random subset of reads from each sample. Each rarefaction curve is labeled by a color corresponding to a specific sample. (b) The first two components are illustrated from a principal component analysis using the log_2_TPM values of the 651 *w*Oo genes analyzed in the study. Points are sized relative to the number of reads mapping to protein-coding genes. The number in parentheses next to each of the axis labels represents the percent variation individually contributed by the two principal components. The heat maps show the (c) TPM values of all protein-coding genes and the (d) z-score of the log_2_TPM values for all protein-coding genes that meet the edgeR expression threshold across all samples in the study. The horizontal bar above each of the heat maps indicates the respective sample for each column.

In our re-analysis, across all samples, between 27,159-153,287 reads map to protein coding genes and a rarefaction analysis shows that all four samples are at/near saturation (Figure 2a, Table S1). A principal component analysis (PCA) clusters the two adult male samples and the two adult female samples in the first principal component, which accounts for 44.5% of the variation (Figure 2b). The second component reveals differences between the male samples that account for 36.7% of the variation, suggesting that the variation observed between the adult male replicates is almost the same as the variation between the adult male and female samples. While hierarchical clustering of the transcript per million (TPM) values (Table S2) of the four replicates again reveals that the adult male and female samples cluster apart, their transcriptome signatures visualized in the heat map are strikingly similar (Figure 2c). No genes are identified as being significantly differentially expressed in edgeR, and while hierarchical clustering resolves males and females, a heat map of the z-score of the log_2_ TPM values illustrates little observed similarity between replicates of males and females, indicating inter-replicate variation is similar to inter-sample variation in this dataset (Figure 2d).

### Luck *et al.*, 2014: The wDi transcriptome in the D. immitis life cycle

*Dirofilaria immitis* is a filarial nematode and the causative agent of heartworm disease, found primarily in canines (McCall *et al.* 2008). A transcriptomics approach was employed to look at the transcriptome at the L3, L4, adult male, adult female, and microfilariae life stages of the *D. immitis* life cycle (Luck *et al.* 2014). Two replicate RNA samples were used for non-stranded library preparation and were sequenced with 50 bp single end Illumina GAIIx reads. In the original analysis, replicates for biological groups were pooled together for transcriptomics analysis, such that there was only one set of counts for each biological sample (Luck *et al.* 2014). Reads were originally aligned using Bowtie (Langmead 2010) before being assembled into transcripts for differential expression analyses using the Cufflinks suite (FDR < 0.01, minimum alignment count: 2) (Trapnell *et al.* 2012) to identify 653 differentially expressed *w*Di genes (Luck *et al.* 2014).

**Figure 3:**
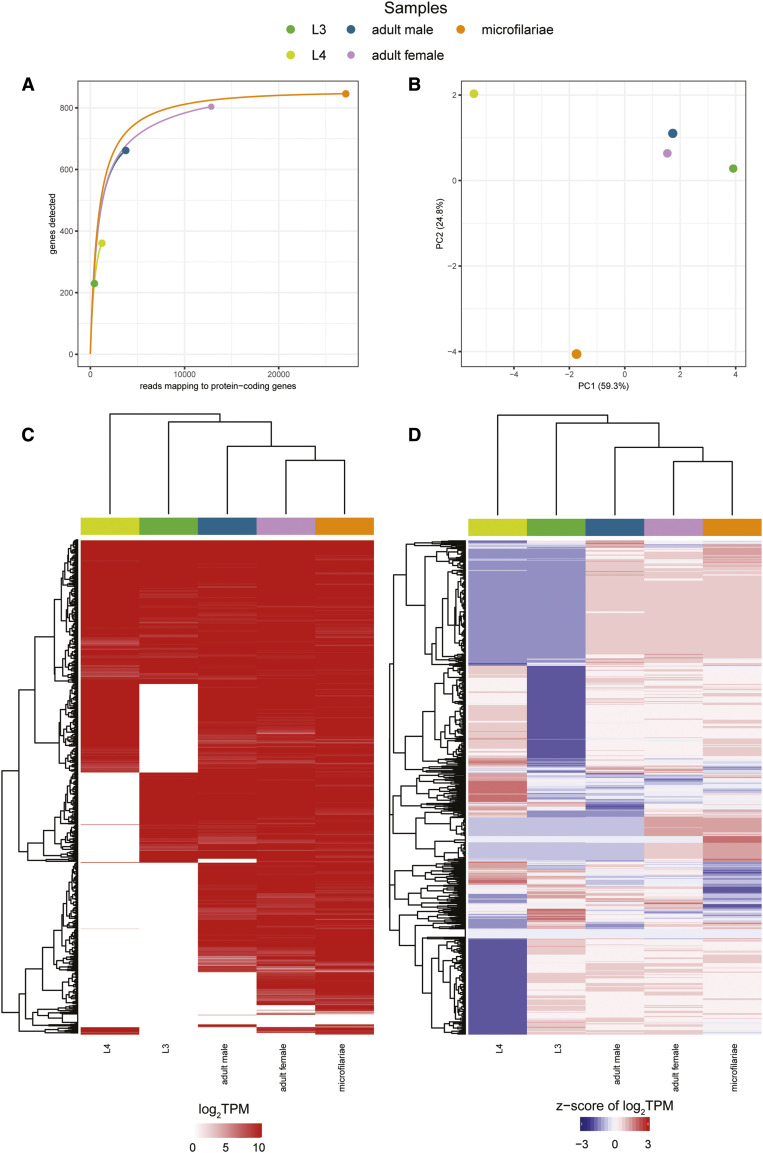
A re-analysis of the Luck *et al.*, 2014 *w*Di life cycle transcriptome study (a) Rarefaction curves were calculated by determining the number of protein-coding genes detected from a random subset of reads from each sample. Each rarefaction curve is labeled by a color corresponding to a specific sample. (b) The first two components are illustrated from a principal component analysis using the log_2_TPM values of the 871 *w*Di genes analyzed in the study. Points are sized relative to the number of reads mapping to protein-coding genes. The number in parentheses next to each of the axis labels represents the percent variation individually contributed by the two principal components. The heat map shows the (c) TPM values for all protein-coding genes and the (d) z-score of the log_2_TPM values for all protein-coding genes across all samples in the study. The horizontal bar above each of the heat maps indicates the respective sample for each column.

Even after pooling in the same manner as in the original study (Luck *et al.* 2014), we obtain an extremely low number of counts for *w*Di genes, ranging from 495-27,118 (Table S1). The low number of mapping reads is likely due to the abundance of host and rRNA transcripts. A rarefaction analysis shows that only the microfilariae samples and adult female samples are at/near saturation (Figure 3a). The PCA does not have an easily interpretable pattern (Figure 3b), and a heatmap of the z-score of the log_2_TPM values illustrates that there are many genes that lack expression values in the L3, L4 and adult male samples (Figure 3c, Table S2). Expression patterns emerge that are an artifact of the lack of reads for so many genes (Figure 3d), and thus we believe that the results reported in the original study need to be reconsidered.

### Darby *et al.*, 2014: The effect of doxycycline exposure on the transcriptome of wMelPop

In the absence of *Wolbachia*, filarial nematodes have defects in development and reproduction, resulting in nematode sterility and eventual death (Slatko *et al.* 2010). The *Aedes albopictus* cell line RML-12 with *w*MelPop-CLA was examined with three replicates with and without exposure to doxycycline hyclate (0.25 ug/mL final concentration) added four days post subculture, with both treated and untreated cells being harvested three days following exposure (Darby *et al.* 2014). Total RNA was extracted, prepared using a stranded RNA-Seq library preparation kit, and sequenced as 100-bp Illumina HiSeq 2000 paired end reads (Darby *et al.* 2014). Reads were originally mapped using BWA (Li and Durbin 2009) and quantified using HTSeq (Anders *et al.* 2015), resulting in 36 and 32 significantly up- and down-regulated genes, respectively, following doxycycline treatment that are involved in a variety of metabolic functions including translation and ribosome assembly; nucleotide, cofactor, and energy metabolism; and DNA replication and transcription (Darby *et al.* 2014).

**Figure 4:**
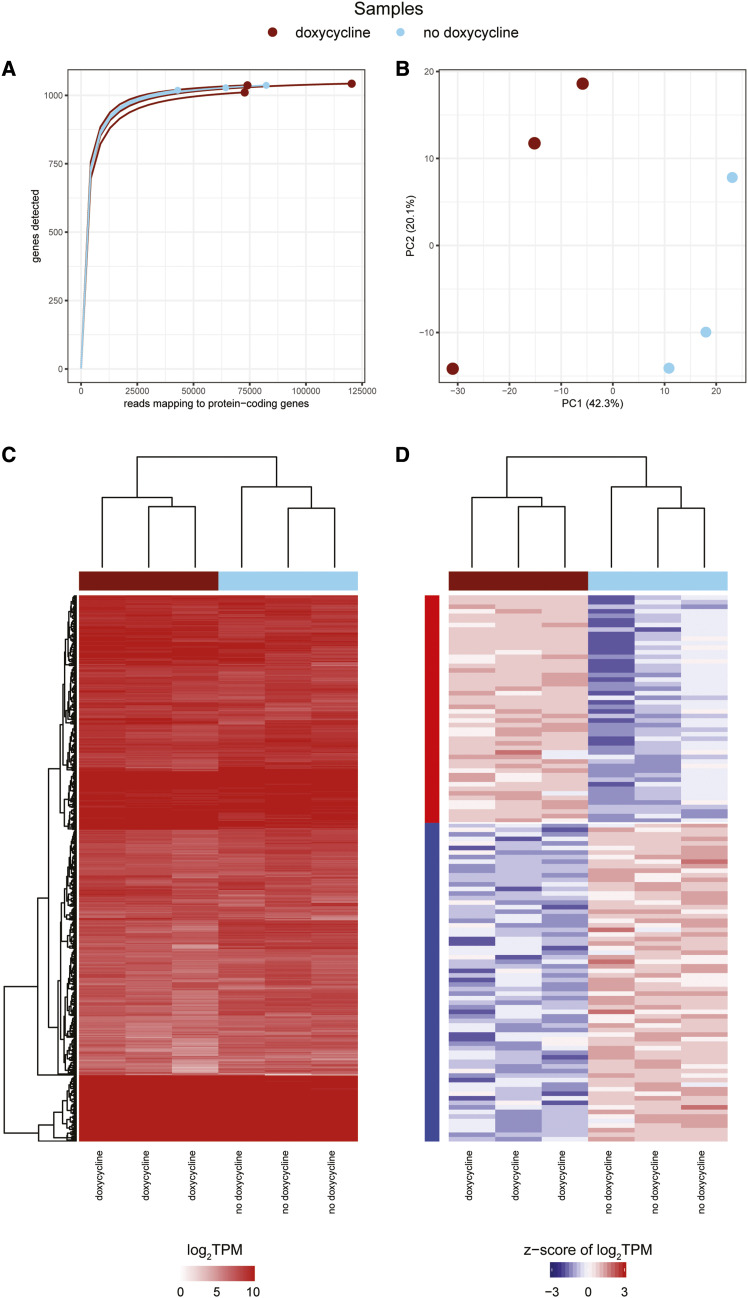
A re-analysis of the Darby *et al.*, 2014 *w*Mel transcriptome study (a) Rarefaction curves were calculated by determining the number of protein-coding genes detected from a random subset of reads from each sample. Each rarefaction curve is labeled by a color corresponding to a specific sample. (b) The first two components are illustrated from a principal component analysis using the log_2_TPM values of the 1,086 *w*Mel genes analyzed in the study. Points are sized relative to the number of reads mapping to protein-coding genes. The number in parentheses next to each of the axis labels represents the percent variation individually contributed by the two principal components. The heat maps show the (c) TPM values for all protein-coding genes passing the read cutoff and (d) the z-score of the log_2_TPM values for all differentially expressed genes across all samples in the study. The horizontal bar above each of the heat maps indicates the respective sample for each column. The left vertical colored bar in (d) represents the WGCNA assigned module.

In our re-analysis, a rarefaction curve of the six samples indicates that all samples are at/near saturation (Figure 4a) (Table S1). A PCA of the six samples shows separation of samples by treatment group in the first principal component (Figure 4b), which is further supported by a hierarchical clustering analysis (Figure 4c, Table S2). We identified 50 and 70, of the 1,086 protein-coding genes, to be differentially expressed up- and down-regulated genes following doxycycline treatment, respectively (Figure 4d, Table S3). No significantly over-represented functional terms were identified in the 50 upregulated genes. The 70 downregulated genes are over-represented in genes with translational functions as structural constituents of the ribosome (Table S4), which was expected (Darby *et al.* 2014) as a well-documented response to ribosomal inhibitors, such as doxycycline (Ng *et al.* 2003; Evers *et al.* 2001).

### Luck *et al.*, 2015: The wDi transcriptome in the different D. immitis tissues

A second *w*Di transcriptomics study examined *Wolbachia* differential expression in the different tissues of *D. immitis* (Luck *et al.* 2015). Total RNA was extracted from each of the tissues, prepared using a non-stranded library preparation kit, and sequenced as 50 bp Illumina GAIIx single-end reads (Luck *et al.* 2015). Any replicates for a given sample were pooled for the transcriptomics analysis (Luck *et al.* 2015). Reads were aligned using Bowtie (Langmead 2010) and assembled into transcripts for several pairwise differential expression analyses using the Cufflinks suite (FDR < 0.01, minimum alignment count: 2) (Trapnell *et al.* 2012). Between the male and female body wall, a total of 33 genes were identified to be differentially expressed. All 33 genes were significantly upregulated in the male body wall samples. Additionally, the female head samples were found to have a similar transcriptional signature to the female body wall samples (Luck *et al.* 2015).

**Figure 5:**
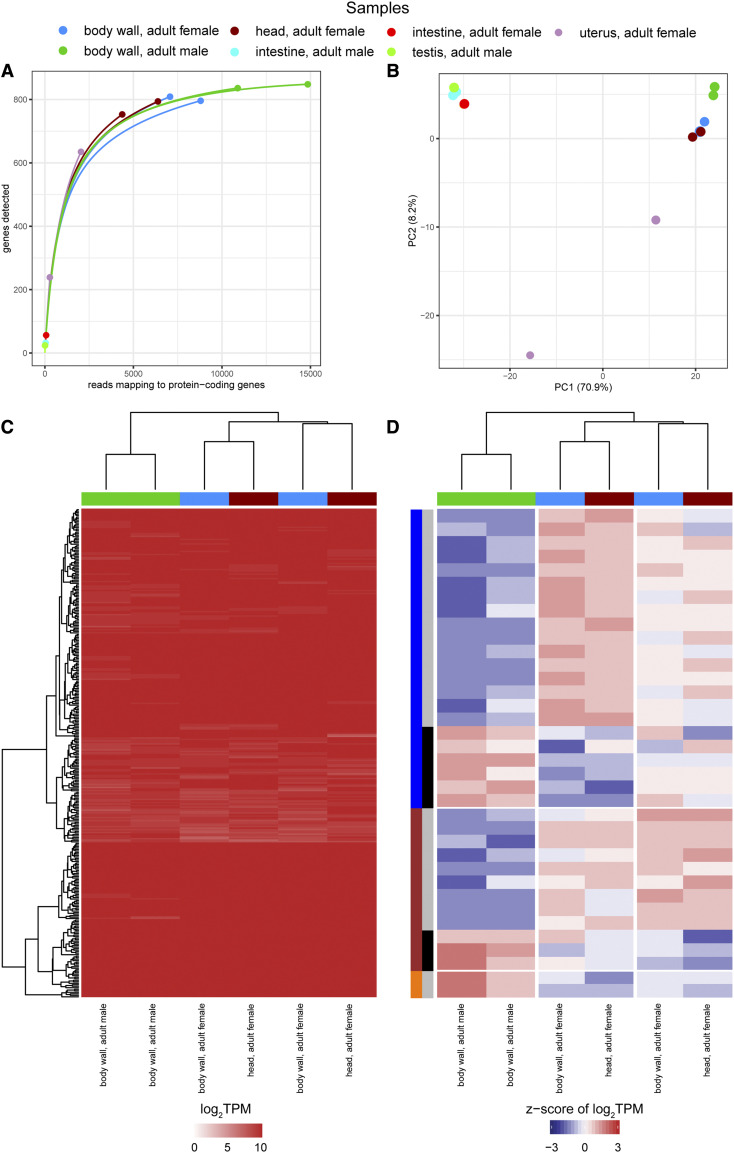
A re-analysis of the Luck *et al.*, 2015 *w*Di tissue transcriptome study (a) Rarefaction curves were calculated by determining the number of protein-coding genes detected from a random subset of reads from each sample. Each rarefaction curve is labeled by a color corresponding to a specific sample. (b) The first two components are illustrated from a principal component analysis using the log_2_TPM values of the 871 *w*Di genes analyzed in the study. Points are sized relative to the number of reads mapping to protein-coding genes. The number in parentheses next to each of the axis labels represents the percent variation individually contributed by the two principal components. The heat maps show the (c) TPM for all protein-coding genes passing the read cutoff and (d) the z-score of the log_2_TPM values for all differentially expressed genes across the adult male body wall, adult female body wall, and adult female head sample, with all other samples being excluded due to having insufficient sequencing depth. The horizontal bar above each of the heat maps indicates the respective sample for each column. The left vertical colored bar in (d) represents the WGCNA assigned module while the right vertical gray scale bar represents the major and minor partitions in each WGCNA module.

Our re-analysis recovers 190-437,866 (average: 174,287) *Wolbachia* reads mapping to protein-coding genes. However, without pooling the samples, a rarefaction analysis shows only six samples to be at/near saturation - both replicates of the adult male body wall, adult female body wall, and adult female head samples (Figure 5a, Table S1, 2). While the original study reported a greater number of reads for each of these samples, we find that most of the *Wolbachia*-mapping reads map to rRNA transcripts (Figure S1). A PCA of the 11 samples analyzed shows that the first principal component separates the samples primarily based on number of reads mapping, while accounting for 70.9% of the total variation observed (Figure 5b). Using only the six samples identified as at/near saturation, 325 genes passed the edgeR minimum expression filter, with 36 genes being identified as differentially expressed across the male and female body wall samples and the female head samples (Figure 5c) that were clustered into three WGCNA expression modules (Figure 5d, Table S3). The adult female head and adult female body wall samples have similar expression profiles, consistent with the original study (Luck *et al.* 2015). Our re-analysis identified 11 genes significantly upregulated in the adult male body wall samples and 25 genes significantly upregulated in the adult female body wall samples with no significantly over-represented functional terms (Table S4).

### Gutzwiller *et al.*, 2015: The wMel transcriptome across D. melanogaster development using modENCODE data

As a part of the modENCODE project (Celniker *et al.* 2009), total RNA was collected across 30 developmental stages of *w*Mel-infected *Drosophila melanogaster* (Gutzwiller *et al.* 2015; Graveley *et al.* 2011), rRNA-depleted, used for stranded library preparation, and sequenced using 100 bp Illumina HiSeq 2000 paired-end reads (Duff *et al.* 2015). Of the 30 life stage samples, 24 had at least two biological replicates and were used for the *w*Mel transcriptomics analysis (Gutzwiller *et al.* 2015). The resulting sequencing reads were mapped with only reverse reads (corresponding to the sense strand) from each read pair being used for quantification to avoid double counting of sequenced fragments. Differential expression analyses were conducted using an ANOVA-like generalized linear model approach with edgeR identifying 80 differentially expressed genes (FDR <0.05). From this, 75 genes were identified as having lower expression levels in the embryo stages relative to either the larval, pupal, and/or adult life stages. The remaining 5 genes show the inverse expression profile, indicating that the entire subset of the 80 *Wolbachia* differentially expressed genes reflect transcriptional changes centered around embryogenesis.

**Figure 6:**
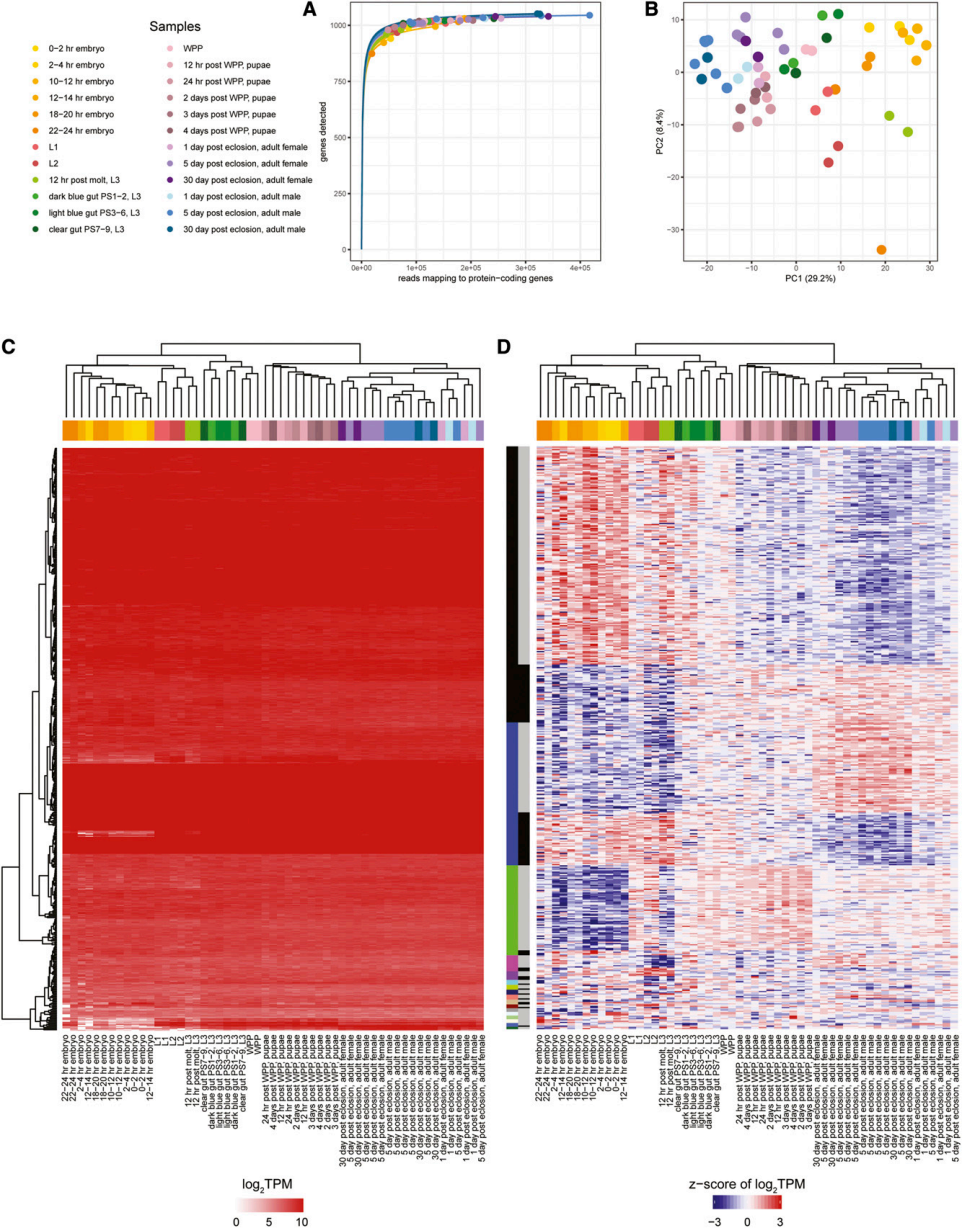
A re-analysis of the Gutzwiller *et al.*, 2015 *w*Mel transcriptome study (a) Rarefaction curves were calculated by determining the number of protein-coding genes detected from a random subset of reads from each sample. Each rarefaction curve is labeled by a color corresponding to a specific sample. (b) The first two components are illustrated from a principal component analysis using the log_2_TPM values of the 1,086 *w*Mel protein-coding genes analyzed in the study. Points are sized relative to the number of reads mapping to protein-coding genes. The number in parentheses next to each of the axis labels represents the percent variation individually contributed by the two principal components. The heat maps show the (c) TPM values for all protein-coding genes passing the read cutoff and (d) the z-score of the log_2_TPM values for all differentially expressed genes across all samples in the study. The horizontal bar above each of the heat maps indicates the respective sample for each column. The left vertical colored bar in (d) represents the WGCNA assigned module while the right vertical gray scale bar represents the major and minor partitions in each WGCNA module.

Our reanalysis of this data set shows that all samples are at/near saturation (Figure 6a, Table S1, 2). A PCA and hierarchical clustering analysis across the 24 different life stage groups shows the samples divided along a continuum of host stages in PC1 with clusters consisting of (a) the post eclosion adult male and adult female samples; (b) the pupae samples post white prepupae (WPP); (c) the embryo samples; (d) the L1, L2, and the 12 hr L3 post molt samples; and (e) the WPP and all other L3 samples (Figure 6bcd) with a transcriptional signature indicative of differences in expression between embryonic stages and the rest of the life cycle as observed in the original study (Gutzwiller *et al.* 2015) (Figure 6cd).

While the original study only identified 80 differentially expressed genes in one or more life stages (Gutzwiller *et al.* 2015), our re-analysis identifies 473 genes as differentially expressed (Table S3). We attribute this difference to an interaction between improper estimation of dispersion in the model and use of the edgeR glmFit method in the previous study, which resulted in lower power in detecting differentially expressed genes. Specifically, the original study omitted the design matrix (specifying which samples are replicates of the same condition) from the dispersion estimation step, resulting in larger common dispersion and larger tagwise dispersion because variation from replicates and conditions were merged together. As a consequence, only a small subset of differentially expressed genes with the highest fold-change were detected in the original study relative to those detected as differentially expressed in the current study (Figure S2). Of the 80 differentially expressed genes identified in the original study (21), 68 are found in the current RefSeq annotation for *w*Mel. Of these 68 genes, 60 genes exceeded the edgeR CPM cutoff implemented here and all 60 of these genes were also identified as differentially expressed in our re-analysis.

There are four major WGCNA-derived clusters with ≥10 genes (Figure 6d, Table S3), with three of these clusters describing an expression profile delineating the embryos and/or larvae samples from the adult and pupae samples. The major partition of the largest expression cluster contains 177 genes upregulated during the embryo life stages and is over-represented in proteins that localize to the cytoplasm and ribosome, including structural constituents of the ribosome (Table S4). The only other cluster identified with over-represented functional terms is the minor partition of the second largest cluster containing 43 genes moderately upregulated in the embryo life stage. This subset of genes contains four of six translation factors with GTP-binding domains identified in the *w*Mel genome, which all encode for proteins with EF-Tu-like domain 2. Additionally, this cluster of genes is also over-represented in ribosomal proteins, including three of the seven proteins that make up the small ribosomal subunit in *w*Mel. This suggests that in the embryos of *D. melanogaster*, the efforts of *Wolbachia* are focused on making translational machinery. This machinery is then poised to be used for translation in the pupae and adult life stages, including the differentially-regulated ones identified in several of the other large clusters (Figure 6d).

### Grote *et al.*, 2017: The wBm transcriptome during the L4 to adult B. malayi life stages

*Brugia malayi* is a filarial nematode and one of the causative agents of lymphatic filariasis and has an obligate relationship with its *Wolbachia* endosymbiont *w*Bm. Using the jird *Meriones unguiculatus* as a model system, the *w*Bm transcriptome was previously analyzed across life stages in the mammalian portion of the *B. malayi* life cycle at the L4, 30 days post infection (dpi), 42 dpi, and 120 dpi timepoints from both male and female *B. malayi* (Grote *et al.* 2017). Each of the seven samples, representing the combinations of life stage and nematode sex, were taken in duplicate and total RNA was used for unstranded library preparations with 150-bp Illumina HiSeq 2500 paired-end reads generated (Grote *et al.* 2017). Reads were mapped to a combined *B. malayi* and *w*Bm reference using Bowtie2 with default settings (Langmead and Salzberg 2012), genes were quantified using HTSeq (Anders *et al.* 2015), and differentially expressed genes were identified using edgeR (FDR < 0.05) (Robinson *et al.* 2010). The original analysis identified only 62 wBm genes differentially expressed across the seven life stages (Grote *et al.* 2017), with the largest number of differentially expressed genes being observed between the 42 dpi and 120 dpi female stages. While genes with roles in a variety of pathways, including oxidative stress, energy production, and DNA replication, were identified as being differentially expressed in *w*Bm during *B. malayi* female development, only the chaperone proteins with roles in protein binding were significantly over-represented in the original study (Grote *et al.* 2017).

**Figure 7:**
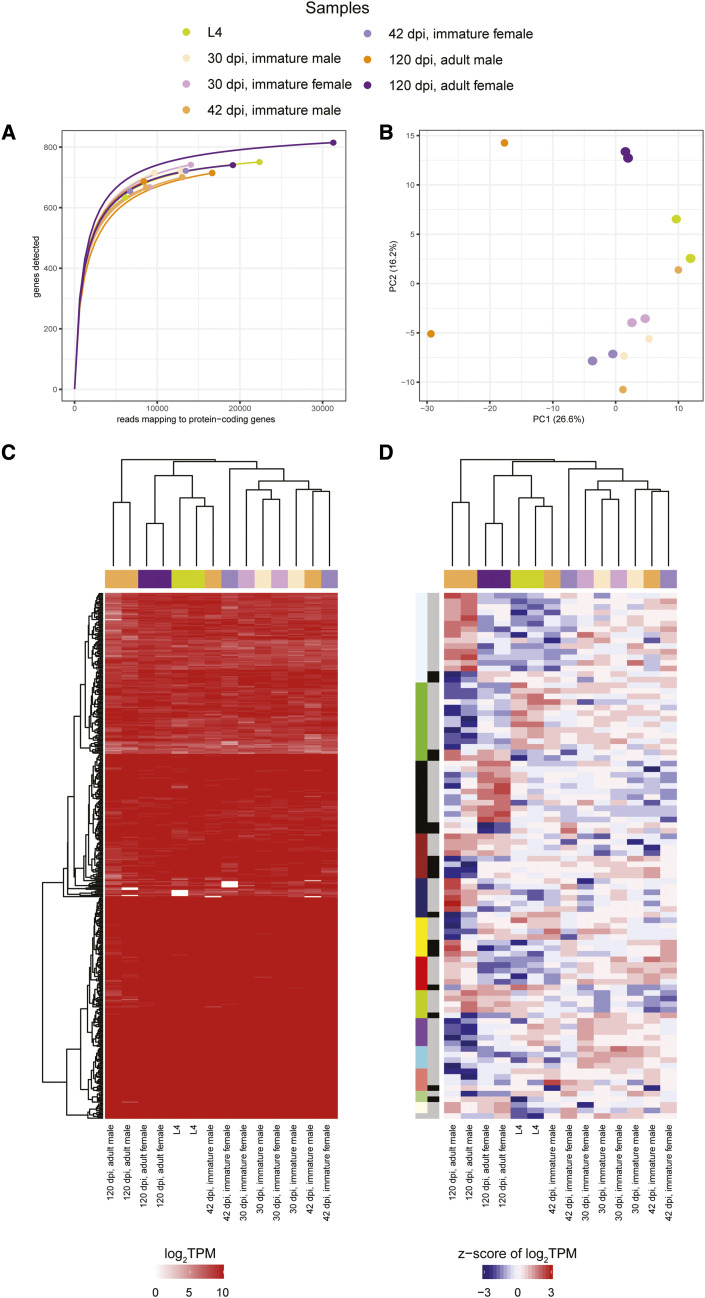
A re-analysis of the Grote *et al.*, 2017 *w*Bm transcriptome study (a) Rarefaction curves were calculated by determining the number of protein-coding genes detected from a random subset of reads from each sample. Each rarefaction curve is labeled by a color corresponding to a specific sample. (b) The first two components are illustrated from a principal component analysis using the log_2_TPM values of the 839 *w*Bm protein-coding genes analyzed in the study. Points are sized relative to the number of reads mapping to protein-coding genes. The number in parentheses next to each of the axis labels represents the percent variation individually contributed by the two principal components. The heat maps show the (c) TPM values for all protein-coding genes passing the read cutoff and (d) the z-score of the log_2_TPM values for all differentially expressed genes across all samples in the study. The horizontal bar above each of the heat maps indicates the respective sample for each column. The left vertical colored bar in (d) represents the WGCNA assigned module while the right vertical gray scale bar represents the major and minor partitions in each WGCNA module.

A rarefaction analysis reveals most of the samples are at/near saturation (Figure 7a, Table S1). A PCA of the seven samples reveals clusters consisting of (a) the L4, 30 dpi and 42 dpi samples regardless of sex, (b) the 120 dpi adult female samples, and (c) the 120 dpi adult male samples (Figure 7b). Additionally, hierarchical clustering of the TPM values shows the 120 dpi adult male and female samples to individually cluster with their replicates while the other samples are more interspersed with one another (Figure 7cd, Table S2).

Our reanalysis of this data set identifies 94 of the 839 protein-coding genes as being differentially expressed. Using WGCNA, we were able to cluster the differentially expressed genes into 14 expression modules, of which only three contained ≥10 genes (Figure 7d, Table S3). We only identified significantly over-represented functional terms in the third largest cluster, which shows 11 genes upregulated at 120 dpi in adult females (Table S4). Similar to the original study (Grote *et al.* 2017), we identify an over-representation of protein folding proteins. Additionally, we find that both *w*Bm genes that encode for portions of the HslUV protease complex are found in this cluster.

### Chung *et al.*, 2019: The wBm transcriptome across the entire B. malayi life cycle

Another study on the transcriptome of *w*Bm was conducted across the complete life cycle of the filarial nematode *B. malayi*, including samples from a mammalian host (*M. unguiculatus*) and an insect vector host (*Aedes aegypti*) (Chung *et al.* 2019). In the vector life stages of filarial nematodes, assessing the *Wolbachia* transcriptome is difficult due to the difference in abundance of both vector and nematode reads relative to *Wolbachia* reads (Choi *et al.* 2014), such that rRNA-, poly(A)-depletions are inadequate in enriching for a sufficient number of *Wolbachia* reads. To address this, Agilent SureSelect RNA-Seq captures were designed for *w*Bm, in order to capture the *w*Bm transcriptional profile for samples with extremely low *Wolbachia* abundance (Chung *et al.* 2018b). Using the Agilent SureSelect capture and rRNA-, poly(A)-depletions, the *w*Bm transcriptome was able to be profiled across the entire *B. malayi* life cycle, consisting of 12 samples from the *B. malayi* mammalian life stages and three samples from the vector life stages. The Agilent SureSelect capture was used for one of the jird 24 dpi immature female samples, two of the jird adult female samples, and all of the vector samples. Total RNA was extracted from at least two biological replicates for each life stage and used to construct stranded RNA libraries to generate 151 bp Illumina HiSeq 4000 paired-end reads (Chung *et al.* 2019). Reads were aligned to a combined *B. malayi* and *w*Bm reference using Bowtie (Langmead 2010) and quantified using FADU with strandedness set to reverse (Chung *et al.* 2018a). A total of 318 genes were identified as differentially expressed using edgeR (FDR < 0.05) (Chung *et al.* 2019) and were clustered into a large number of poorly defined WGCNA expression modules (Chung *et al.* 2019). The largest module consisted of 51 genes differentially expressed in the vector life stages, but was not enriched for any specific functional terms. The poorly defined nature of the recovered gene clusters supports minimal transcriptional regulation, potentially due to the limited environmental variation for obligate intracellular bacteria (Chung *et al.* 2019).

**Figure 8:**
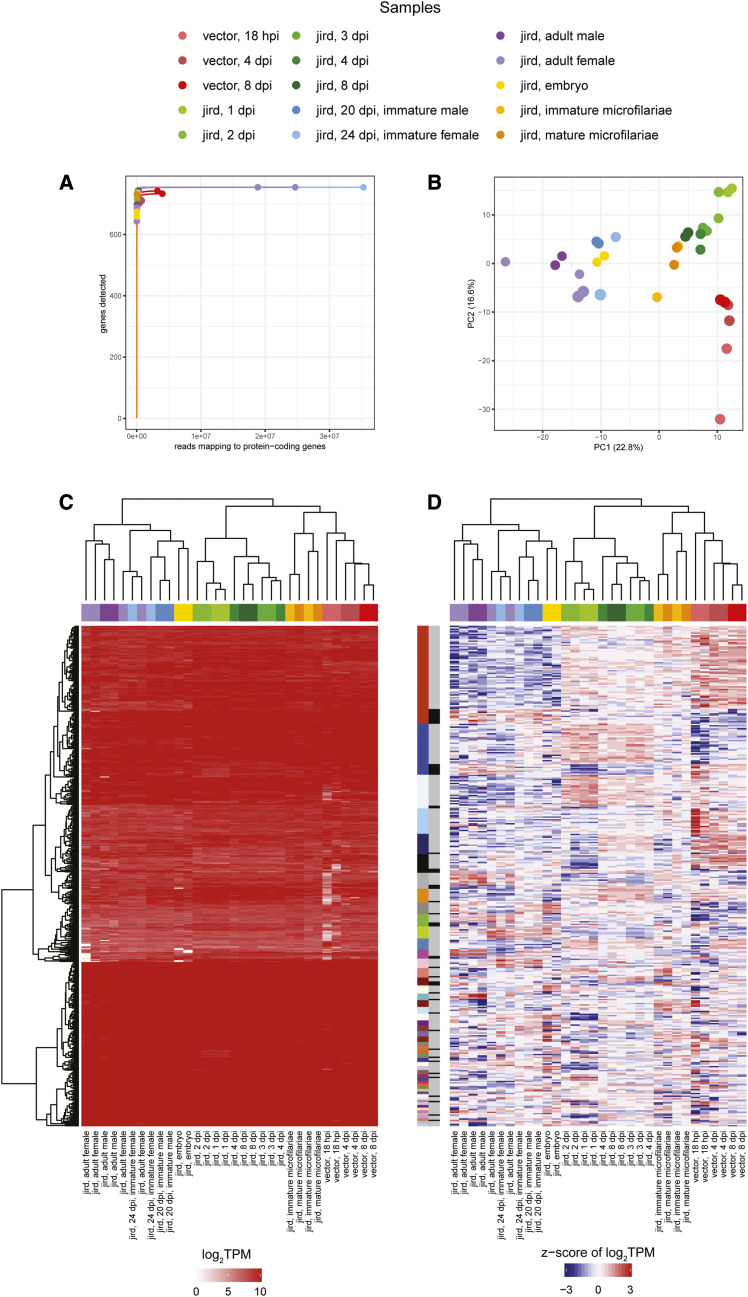
A re-analysis of the Chung *et al.*, 2019 *w*Bm transcriptome study (a) Rarefaction curves were calculated by determining the number of protein-coding genes detected from a random subset of reads from each sample. Each rarefaction curve is labeled by a color corresponding to a specific sample. (b) The first two components are illustrated from a principal component analysis using the log_2_TPM values of the 839 *w*Bm genes analyzed in the study. Points are sized relative to the number of reads mapping to protein-coding genes. The number in parentheses next to each of the axis labels represents the percent variation individually contributed by the two principal components. The heat maps show the (c) TPM for all protein-coding genes passing the read cutoff and (d) the z-score of the log_2_TPM values for all differentially expressed genes across all samples in the study. The horizontal bar above each of the heat maps indicates the respective sample for each column. The left vertical colored bar in (d) represents the WGCNA assigned module while the right vertical gray scale bar represents the major and minor partitions in each WGCNA module.

A rarefaction analysis shows all samples are at/near saturation with five samples, all prepared using the Agilent SureSelect enrichment, having a much greater number of reads: (a) one of the jird 24 dpi immature female samples, (b) two of the jird adult female samples, and (c) two of the vector 8 dpi samples (Figure 8a, Table S2). A PCA shows that the vector samples separate from the mammalian samples, with the vector samples clustering most closely with the microfilariae and mammalian L3 samples (1, 2, 3, 4, 8 dpi) in the first principal component (Figure 8b). One concern was that the use of the Agilent SureSelect enrichment would significantly bias the *Wolbachia* transcriptome relative to the recovered transcriptome from the poly(A)-, rRNA-depleted libraries. After excluding 85 protein-coding genes that had inadequate coverage in the Agilent SureSelect probe design, we observed a hierarchical clustering pattern that indicates samples are grouping by life stage rather than library preparation, as desired (Figure 8c). A differential expression analysis indicates that of the remaining 754 protein-coding genes, 373 are differentially expressed compared to the 318 identified in the original study (Figure 8d) (Chung *et al.* 2019) with 8 WGCNA expression clusters with ≥10 genes (Figure 8d, Table S3).

Of the 8 expression clusters, only 2 contained genes significantly over-represented in a specific functional term. In the largest cluster, describing *w*Bm genes highly upregulated in the vector life stages and moderately upregulated in the microfilariae and the mammalian L3 life stages, which are all early larval stages for *B. malayi*, we find 62 upregulated genes that are significantly over-represented in ribosomal and translational proteins (Table S4). In the fifth largest module, indicating genes specifically upregulated in the vector life stages, we also observe an over-representation of proteins with GTPase activity.

### Identifying shared differentially expressed genes across all data sets

**Figure 9:**
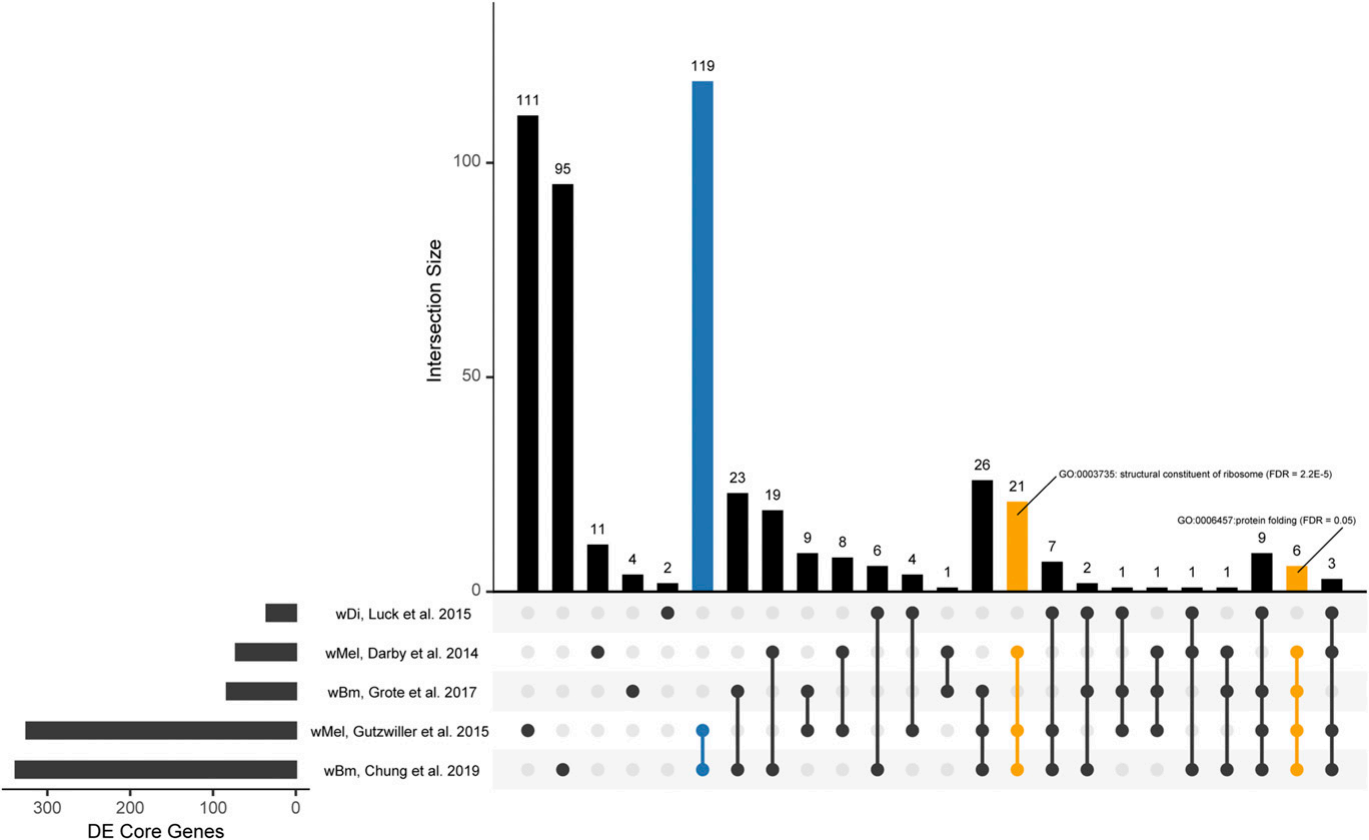
UpSet plot of core *Wolbachia* genes across transcriptomics data sets An UpSet plot was generated from the differentially expressed core *Wolbachia* genes from each study. The bottom left shows a horizontal bar plot indicative of the number of differentially expressed core *Wolbachia* genes identified in our re-analysis. The larger vertical bar plot indicates the number of differentially expressed core genes in the studies indicated by the nodes under the x-axis. Yellow bars are indicative of gene subsets that contain significantly over-represented functional terms while blue bars are indicative of gene subsets that contain genes of interest.

Using the differentially expressed genes recovered from each study, we assessed whether there were core *Wolbachia* genes consistently differentially expressed across the 5 studies with recovered differentially expressed genes. A core genome analysis conducted using PanOCT (Fouts *et al.* 2012) between the *w*Bm, *w*Di, *w*Mel, and *w*Oo genomes recovered 640 core genes present as single copy orthologs in each genome (**Table S6**). Across all studies, between 60–97% of recovered differentially expressed in the re-analysis were identified to be core genes (Table 1). An UpSet analysis (Lex *et al.* 2014; Conway *et al.* 2017) was used to separate the differentially expressed genes into intersections based on the number of similarly differentially expressed core genes (Figure 9). For each intersection, we conducted a functional term enrichment analysis to identify consistent biological processes differentially regulated by the *Wolbachia* across different systems (FDR < 0.05) (Table S7).

The Gutzwiller *et al.*, 2015 and Chung *et al.*, 2019 studies share 119 differentially expressed core genes, the most across all intersections (Figure 9). While a functional term enrichment analysis recovers no significantly over-represented biological process in this 119 gene subset, we find 8 of 15 genes encoding proteins containing RuvA domain 2-like domains, known to play a role in homologous recombination (Badawi *et al.* 2014). Similarly, this intersection contains 5 of 7 genes encoding for proteins with a NAD-dependent DNA ligase BRCT domain., another protein domain known to have a role DNA recombination and repair (Shrivastava *et al.* 2012).

Of the remaining intersections, only two have recovered significantly over-represented functional terms from the enrichment analysis (Figure 9). The 21 core genes shared between the Darby *et al.*, 2014; Gutzwiller *et al.*, 2015, and Chung *et al.*, 2019 studies are over-represented in genes encoding structural constituents of the ribosome (FDR < 2.2E-5). While this may be expected from the doxycycline treatment conditions used in the Darby *et al.* study, the Chung *et al.* study finds these ribosomal proteins to be differentially expressed in the early *B. malayi* larval stages while the Gutzwiller *et al.* study finds them to be differentially expressed in only the embryo life stage of *D. melanogaster*.

The other intersection with recovered significantly over-represented functional terms contains 6 core genes differentially expressed in the Darby *et al.*, 2014; Gutzwiller *et al.*, 2015, Grote *et al.*, 2017, and Chung *et al.*, 2019 studies. Of these 6 core genes, 4 have protein folding roles (FDR = 0.05). While the Gutzwiller *et al.* study finds them to be differentially expressed in the embryos while the Grote *et al.* and Chung *et al.* studies find them to be differentially expressed in the adult female or adult life stages, respectively. While this highlights differential expression at different timepoints between arthropod and nematode *Wolbachia*, these results indicate a commonality in the differential regulation of ribosomal and protein folding proteins across all *Wolbachia*.

## Discussion

### A common pan-Wolbachia transcriptional response

Of the seven studies, our re-analysis identifies <100 differentially expressed genes from the Darby *et al.*, 2012; the Luck *et al.*, 2015; and the Grote *et al.*, 2017 studies. This may suggest that *Wolbachia* endosymbionts largely lack global gene regulation and instead constitutively express a vast majority of their genes while regulating only a small number of genes. Lack of global gene regulation was proposed in the analysis of both the *w*Bm (Foster *et al.* 2005) and *w*Mel genomes (Wu *et al.* 2004), in which few transcriptional regulators were identified. The intracellular niche where *Wolbachia* reside is relatively static, possibly explaining the general lack of differential expression in *Wolbachia*. Similarly, in the bacterial genus *Buchnera*, which consists of aphid endosymbionts, little regulation has been observed to occur at the transcriptional level, with regulation occurring primarily at the post-translational level through translational inhibition and activation along with transcript stability (Hansen and Degnan 2014). This may be a trait that is more universal among intracellular bacteria, including many important human pathogens. However, this may be surprising for nematode *Wolbachia*, given that their nematode hosts move between an invertebrate cold-blooded host and a vertebrate warm-blooded host given that such temperature shifts lead to sigma factor-dependent regulation in many other bacteria. However, it is possible that loss of temperature-induced sigma factor-dependent regulation was lost in the lineage prior to the divergence of the filarial nematode *Wolbachia* strains.

For our re-analysis of the two largest datasets (Gutzwiller *et al.*, 2015 and Chung *et al.*, 2019), we observe >35% of *w*Mel and *w*Bm genes to be differentially expressed, respectively. The relatively large number of *w*Mel genes identified in our re-analysis differs from the results in the original study by Gutzwiller *et al.*, 2015. This difference is attributable to improved sensitivity to detect differentially expressed genes resulting from improved modeling methods used in the current study. Applying this common analytical framework across datasets reveals a consistent upregulation of *Wolbachia* genes involved in ribosome biosynthesis and translation in early life stages like embryos in wMel and larva in wBm relative to adult life stages. Remarkably, this pattern is consistently observed across these two diverse *Wolbachia* endosymbionts with hosts that span Insecta and Nematoda. These and all other *Wolbachia* hosts are included in the Ecdysozoa, which are united by undergoing periodic molting (ecdysis) (Telford *et al.* 2008), and thus this conserved *Wolbachia* transcriptional response may be primarily stimulated by host pathways involved in molting. Beyond this commonality, the additional major expression modules recovered from the Gutzwiller *et al.* study are largely centered on the differential expression observed at the embryo life stage, with no other significantly over-represented functional terms being identified. Additionally, both the Gutzwiller *et al.* and Chung *et al.* studies recover smaller and more numerous expression modules, possibly indicative of noisy transcriptional signatures or species-specific regulation. Further transcriptomics studies of *Wolbachia* across life cycle stages of other hosts with additional replicates are needed to resolve these possibilities.

### Re-evaluation of early dual species transcriptomics datasets

Because *Wolbachia* endosymbionts are obligate intracellular bacteria that in some stages are at very low density, sufficiently sequencing the *Wolbachia* transcriptome for differential expression analyses is technically challenging and often cost prohibitive. Using this unified analysis pipeline, derived from current best practices for transcriptomics analyses, allows us to both critically re-evaluate and identify novel expression patterns in older *Wolbachia* transcriptomics studies. The application of saturation curves to each of these studies has shown that the earliest *w*Di transcriptome study did not have sufficient sequencing depth to be biologically informative. Implementing the counts per million (CPM) cut-offs that are currently recommended in the edgeR manual (Robinson *et al.* 2010), ensures that each gene has a sufficient number of reads for analysis. In dual species analyses, where it may be difficult to get many reads or ensure the same number of reads in every sample, it is important that a CPM cut-off be implemented in a way that avoids the biased influence of samples with more sequencing reads. Additionally, applying more modern and sensitive differential expression analyses tools have allowed us to identify novel *w*Bm and *w*Mel expression patterns not identified in the original studies (Gutzwiller *et al.* 2015; Grote *et al.* 2017; Chung *et al.* 2019).

### Research parasitism, secondary data analysis, and meta-analyses

Research parasite is a “tongue in cheek” term coined to describe scientists who reuse publicly available data for secondary data analysis (Longo and Drazen 2016), including meta-analyses like this one. Research parasitism has been enabled through advocacy by researchers, funding agencies, and journals to require deposition of raw, unfiltered sequencing data in public repositories like the NCBI sequence read archive. One of the major *Wolbachia* transcriptome datasets used here, and in other studies (Lindsey *et al.* 2018; LePage *et al.* 2017), exemplifies research parasitism by relying on *Drosophila* transcriptome data made publicly available by the modENCODE project (Celniker *et al.* 2009; Duff *et al.* 2015; Gutzwiller *et al.* 2015).

Without the deposition of data for each of the studies examined here, this meta-analysis would not have been possible. By reanalyzing expression data using a common pipeline, we can identify discrepancies between the re-analysis and original studies, isolate their causes, and have confidence that the observed common biological signatures across datasets in our study are not due to differences in analytical methods. Thus, the new biological results drawn from this analysis could only be made through secondary analysis and research parasitism. This was also facilitated by a strong, collaborative *Wolbachia* community that came together to facilitate this re-analysis in a constructive manner.

## Conclusions

Our re-analysis of the available *Wolbachia* transcriptomics data sets identified a coordinated transcriptional response of translational proteins across diverse *Wolbachia* strains and host contexts. This study also demonstrates the importance of depositing raw sequencing data, as this meta-analysis would not have been possible without this resource sharing as well as the collaborative nature of the *Wolbachia* community.
